# Facile synthesis of rGO/NiWO_4_ hybrid electrocatalyst for enhanced oxygen evolution reaction in alkaline medium

**DOI:** 10.3389/fchem.2026.1799700

**Published:** 2026-04-28

**Authors:** D. J. Patil, D. B. Malavekar, V. C. Lokhande, J. H. Kim, C. D. Lokhande

**Affiliations:** 1 Centre for Interdisciplinary Research, D. Y. Patil Education Society (Deemed to Be University), Kolhapur, India; 2 Department of Applied Science and Humanities, TKIET, Warana University Warananagar, Kolhapur, India; 3 Department of Materials Science and Engineering, Chonnam National University, Gwangju, Republic of Korea; 4 Energy storage research group, School of Chemistry and Physics, Queensland University of Technology, Brisbane, QLD, Australia

**Keywords:** nickel tungstate, oxygen evolution reaction, reduced graphene oxide, successive ionic layer adsorption and reaction, water splitting

## Abstract

Growing energy consumption and concerns regarding the effects of global warming are driving development of greener energy alternatives. Green hydrogen production and consumption are essential in modern energy conversion and storage. The progression of water splitting technologies for sustainable hydrogen production relies on the development of efficient and cost-effective electrocatalysts for the oxygen evolution reaction (OER). Here, we present nickel tungstate (NiWO_4_) and reduced graphene oxide composited nickel tungstate (rGO/NiWO_4_) synthesis via an easily processable successive ionic layer adsorption and reaction (SILAR) method, and its OER performance in an alkaline electrolyte. While NiWO_4_ exhibited poor OER performance and stability, the incorporation of rGO significantly stabilized the composite electrode and extended its stability. The introduction of rGO reduced the charge transfer resistance and enhanced the surface area of the composite electrode compared to NiWO_4_. As a result, rGO/NiWO_4_ electrocatalyst exhibited a lower overpotential of 210 ± 10 mV at 50 mA cm^−2^ and a Tafel slope of 60 ± 3 mV dec^−1^, compared to NiWO_4_ (260 ± 13 mV, 60 ± 3 mV dec^−1^), showing enhanced catalytic performance. Additionally, the composite electrode demonstrated long term stability, over 50 h of continuous operation. These findings demonstrate rGO/NiWO_4_ as a promising OER electrocatalyst for efficient water splitting.

## Graphical Abstract

**Figure:**
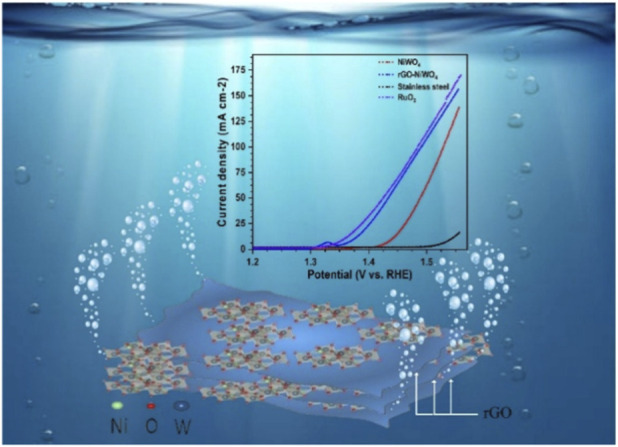

## 1 Introduction

The profound environmental challenges stemming from contemporary energy utilization have induced a state of irreversible climate change. In response, there is a rapid shift towards alternative energy sources, notably wind turbines, solar photovoltaic systems, and hydrogen. Given the intermittent nature of solar and wind energy, energy storage has become a pivotal area of scholarly investigation and technological advancement (Mirkin et al., 2024; Worku et al., 2024). Various storage methodologies, including electrochemical storage and pumped hydro storage, are being developed, alongside conversion technologies such as hydrogen generation (Raza S. et al., 2024). Hydrogen, specifically, is recognized as feasible green fuel for increased uptake (Jin et al., 2022; Sosa et al., 2021). Electrocatalytic water splitting, which converts the electricity generated by other renewables into hydrogen, holds immense potential (Tiwari et al., 2023). A crucial aspect of this research involves the synthesis of innovative electrocatalytic materials and the comprehensive evaluation of their electrochemical properties to determine their suitability for water electrolysis applications. Platinum (Pt), Ruthenium (Ru), Palladium (Pd), and Iridium (Ir)-based materials used are cutting edge materials for water splitting (Gaikwad et al., 2024; Malavekar et al., 2024). However, their widespread application is constrained by excessive expenses and scarcity. Consequently, there is an intensive effort among researchers to develop electrocatalysts using earth abundant materials. Enhancing catalytic efficiency is pursued through various strategies of synthesis of material (Malavekar et al., 2022).

Various synthesis approaches have been employed to enhance electrocatalytic ability of materials, including the synthesis of multivalent electrode materials, doping techniques, morphology engineering, preparation of heterostructure materials, formation of crystalline amorphous materials, and development of self-supported architectures (Lokhande et al., 2024) (Raza A. et al., 2024; Zheng et al., 2024). These strategies have demonstrated success in achieving electrocatalytic performance suitable for industrial scale applications. Nickel-based materials, in particular, have shown promising activity for oxygen evolution reactions (OER) in water splitting applications. Various forms of nickel compounds, such as NiO, NiOOH, Ni(OH)_2_, Ni_2_P, NiMoO_4_, NiWO_4_, among others, have been synthesized and investigated (Jin et al., 2021; Molla et al., 2023; Sun et al., 2024). For instance, a heterostructure composed of Ni(OH)_2_/Ni_3_S_2_ hybrid nanosheet arrays, fabricated through a two-step co-precipitation and sulfuration method by Du et al. (Du et al., 2018) Recent studies highlight the strong performance of certain electrocatalysts for the OER. For instance, one system was able to achieve a current density of 20 mA cm^−2^ at a relatively low overpotential of 211 mV, underscoring its efficiency. Similarly, Fe-doped Ni_2_P nanosheet arrays have emerged as promising bifunctional catalysts, showing effectiveness not only for OER but also for overall water splitting (Du et al., 2018). In another approach, cobalt and nickel based nanotube structures demonstrated notable catalytic activity, reaching 10 mA cm^−2^ at an overpotential of 250 mV (Du et al., 2018). Among the wide range of candidates explored, nickel-based materials stand out as particularly powerful OER electrocatalysts, and their development has been the focus of extensive research efforts. In addition, amorphous materials are pivotal in catalytic reactions, offering distinct advantages over crystalline counterparts. The amorphization process represents a straightforward and highly effective strategy to introduce crystalline defects and enhance short range ordering within catalyst materials (Chen et al., 2021). Owing to their chemical and structural disorder, amorphous catalysts exhibit superior performance compared to crystalline counterparts, showcasing improved electrochemical stability and intrinsic activity (Gupta et al., 2019; Zhou and Fan, 2021).

This study used the successive ionic layer adsorption and reaction (SILAR) method to synthesize amorphous NiWO_4_ on stainless steel (SS) substrates. To enhance the specific surface area and effectively modulate the active centre density of the electrode material, reduced graphene oxide (rGO) was introduced into NiWO_4_. The resulting heterostructured material exhibited exceptional electrocatalytic performance for the OER, attributed to synergistic effects between rGO and NiWO_4_. Electrochemical impedance spectroscopy (EIS) was employed to investigate the charge transfer kinetics, providing valuable insights into the behavior and performance of rGO-NiWO_4_ thin films during OER. This study establishes a foundation for the design, synthesis, and electrochemical applications of cost-effective Ni-based electrocatalysts.

## 2 Experimental

### 2.1 Chemicals and materials

Chemical synthesis was carried out using analytical grade (AR) chemicals. Chemicals such as NiSO_4_·6H_2_O (Purity 99%), Na_2_WO_4_·2H_2_O (Purity 99%), and KOH (Purity 85%) were utilized for synthesis and electrochemical measurements. The double distilled water (DDW) served as the solvent. Before deposition, a 0.5 mm thick 304-grade SS substrate was cleaned adopting the following procedure. The SS substrates were cut into dimensions of 1 cm × 4 cm and mechanically polished using 2000-grade silicon carbide sandpaper. Subsequently, the substrates were ultrasonically cleaned in DDW for 10 min, gently swabbed with cotton to remove residual impurities, and then dried at room temperature.

### 2.2 Development of NiWO_4_ and rGO-NiWO_4_ electrodes

NiWO_4_ thin films were prepared using the SILAR technique. In this process, aqueous solutions of 0.1 M Na_2_WO_4_·2H_2_O and 0.1 M NiSO_4_·6H_2_O served as the sources of tungstate (WO_4_^2−^) and nickel (Ni^2+^) ions. The deposition began by immersing SS substrate in the nickel precursor solution for 20 s, followed by rinsing with DDW for 10 s to wash away weakly adsorbed Ni^2+^ ions. The substrate was then dipped into the tungstate solution for another 20 s. At this stage, the Ni^2+^ ions pre-adsorbed on the surface reacted with WO_4_^2−^ ions, leading to the formation of insoluble NiWO_4_ directly on the SS substrate. A final rinse in DDW for 10 s ensured the removal of any loosely attached NiWO_4_ nanoparticles, resulting in a uniform thin film. A total of 100 cycles were performed to obtain the optimized electrode material on the SS substrate.

rGO was prepared following protocol reported in our previous report (Malavekar et al., 2020). To prepare rGO suspension of concentrations of 10 mg mL^−1^, optimised quantity of rGO was mixed in the DDW and sonicated for 6 h. Layered deposition of rGO sheets over a SS substrate was followed by the SILAR deposition of NiWO_4_ nanoparticles. For deposition of rGO-NiWO_4_ electrode, a 10 mg mL^−1^ rGO solution was placed before the first beaker containing NiSO_4_·6H_2_O solution used for NiWO_4_ electrode deposition, making five beaker synthesis process. The SS substrate was immersed in rGO solution for 30 s, followed by the same deposition process as NiWO_4_. This process was repeated for 100 cycles for a comparative study. For the above described deposition process, automated SILAR system was employed. The characterization data, which substantiate the structural and functional properties of the materials, are provided in the Supporting Information.

## 3 Results and discussion

### 3.1 Growth mechanism of NiWO_4_ and rGO-NiWO_4_ electrodes

**FIGURE 1:**
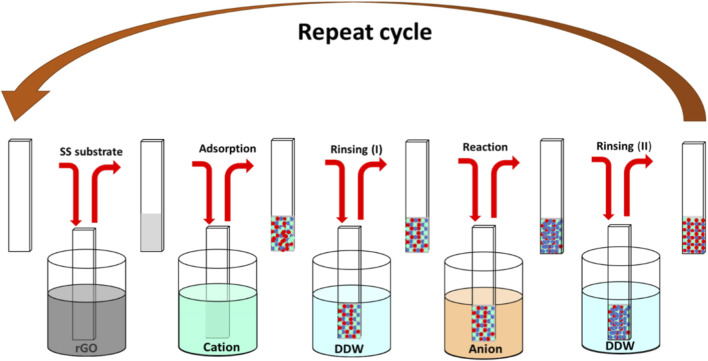
The SILAR method schematic used to prepare rGO/NiWO_4_ thin-film electrodes.

The NiWO_4_ and rGO-NiWO_4_ electrodes were synthesized using a SILAR method employing a four and five beakers setup, respectively (Figure 1). For rGO-NiWO_4_, the first beaker contained a well dispersed rGO solution of density 10 mg mL^−1^. The second beaker contend 0.1 M NiSO_4_·6H_2_O solution that provides Ni^2+^ ions and controls the adsorption rate. The fourth beaker is of an anionic precursor Na_2_WO_4_·2H_2_O controlling reaction rate. The third and fifth beakers contained DDW for rinsing purposes to prevent excessive surface aggregation and ensure uniform film growth. The dissociation of NiSO_4_·6H_2_O (Equation 1) and Na_2_WO_4_·2H_2_O (Equation 2) in aqueous solution can be represented by the following reactions:

$${\text{NiSO}}_{4}\cdot 6H_{2}O\;↔\;{\text{Ni}}^{2+}+{\left({\text{SO}}_{4}\right)}^{2-}+6H_{2}O$$ (1)

$${\text{Na}}_{2}{\text{WO}}_{4}\cdot 2H_{2}O\;↔\;2{\text{Na}}^{+}+{\left({\text{WO}}_{4}\right)}^{2-}+2\;H_{2}O$$ (2)

During the deposition process, the SS substrate was initially immersed in rGO suspension which was prepared using the process reported in our previous articles. The rGO sheets were physically adsorbed on the substrate after immersion. The negatively charged rGO sheets layer facilitate an excellent adsorption platform for Ni^2+^ ions onto its surface. Upon subsequent immersion in Na_2_WO_4_·2H_2_O solution, a chemical reaction between the pre-adsorbed Ni^2+^ ions and (WO_4_)^2−^ ions resulted in the formation of a composite rGO-NiWO_4_ electrocatalyst. During the growth of NiWO_4_, ion-by-ion deposition mechanism, where nucleation was initiated by the adsorption of Ni^2+^ ions at nucleation centres on the immersed substrate. The layer-by-layer growth process ensured the uniform formation of rGO-NiWO_4_ film.

### 3.2 Physico-chemical characterisations

**FIGURE 2:**
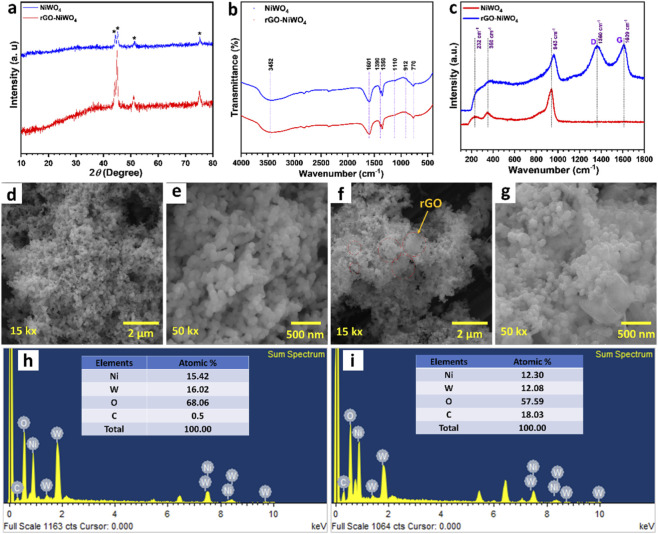
**(a)** XRD patterns, **(b)** FT-IR spectra, **(c)** Raman spectra of NiWO_4_ and rGO-NiWO_4_ composite materials. The SEM images of **(d,e)** NiWO_4_, and **(f,g)** rGO-NiWO_4_ composite material at magnifications of 15kX and 50kX. The EDS spectra of **(h)** NiWO_4_, and **(i)** rGO-NiWO_4_ composite materials (inset shows atomic percentages of Ni, W, O, and C elements).

The crystal structure and lattice parameters of the NiWO_4_ and rGO-NiWO_4_ were analysed using the XRD. The XRD patterns of NiWO_4_ and rGO-NiWO_4_ electrodes are shown in Figure 2a. The XRD pattern exhibits no distinct diffraction peaks other than those originating from the SS substrate, confirming the amorphous nature of the deposited material both before and after rGO incorporation. The formation of the amorphous structure can be attributed to the room-temperature synthesis conditions employed during the growth process on the SS substrate. The peaks marked with an asterisk (*) correspond to the diffraction peaks of the SS substrate.

FT-IR spectroscopy was used to study the bonding characteristics of NiWO_4_ and its rGO–NiWO_4_ composite (Figure 2b). A broad absorption band between 3115 and 3550 cm^−1^ corresponds to–OH stretching vibrations, suggesting the presence of adsorbed water molecules. The bending vibration of the–OH group is evident at 1588 cm^−1^, while additional peaks at 1364 and 1384 cm^−1^ are also linked to–OH stretching. Absorption bands at 1064 and 929 cm^-1^ indicate asymmetric W–O–W stretching, and the band at 801 cm^−1^ is associated with terminal W–O–W bonds in WO_6_ octahedra. A sharp peak at 572 cm^−1^ arises from symmetric Ni–O stretching, confirming the successful formation of NiWO_4_ on the SS substrate. Although no new peaks appear in the rGO–NiWO_4_ composite, the overall absorption intensity is enhanced compared to pure NiWO_4_.

Raman spectroscopy (Figure 2c) provided further insights into the molecular structure of the prepared materials. For pristine NiWO_4_, characteristic Raman peaks were observed at 930 cm^−1^ corresponding to O–W–O stretching vibrations, 347 cm^−1^ associated with the bending modes of WO_6_ octahedra, and 221 cm^−1^ attributed to O–Ni–O stretching vibrations, confirming the formation of NiWO_4_. In the rGO–NiWO_4_ composite, additional Raman bands appeared at 1339 cm^−1^ and 1609 cm^−1^, corresponding to the D and G bands of rGO, respectively, which confirms the successful incorporation of rGO into the composite. The intensity of the D band being higher than that of the G band suggests the presence of structural defects and the effective reduction of graphene oxide. Furthermore, the Raman peaks associated with NiWO_4_ exhibited reduced intensity and a slight shift, with a prominent O–W–O stretching peak observed at 943 cm^−1^ compared to pristine NiWO_4_, indicating strong interaction between rGO and NiWO_4_. A similar shift in the rGO bands was also observed when compared with the Raman spectrum of pure rGO (Supplementary Figure S1), where the D and G bands appear at 1345 and 1582 cm^−1^, respectively. The shift of these peaks toward higher wavenumbers after composite formation suggests electronic interaction between rGO and NiWO_4_.

Microstructure of NiWO_4_ and rGO-NiWO_4_ composite materials were investigated using SEM at 15kX and 50kX magnifications, and corresponding SEM images are shown in Figures 2d–g. NiWO_4_ showed spherical particles connected to each other forming porous structure while the morphology in rGO-NiWO_4_ remains same additional rGO sheets were observed, highlighted in circle. These rGO sheet acts as a spacer, abrupting NiWO_4_ particle growth. Due to this, the intensity of peaks in XRD decreased in composite material. Such structure can facilitate the advantage of catalysis through NiWO_4_ and facile charge transfer through rGO. The particle size distribution analysis revealed no significant change in the average particle size of NiWO_4_ after rGO incorporation. Both pristine NiWO_4_ and the rGO-NiWO_4_ composite exhibited a similar average particle size of approximately ∼120 nm (Supplementary Figure S2). However, the particle size distribution indicates that the range of particle sizes becomes broader upon the introduction of rGO. Additionally, the EDS analyses of NiWO_4_ and rGO-NiWO_4_ composite materials are provided in Figures 2h,i, respectively. The inset of Figure 2h presents the atomic percentages of Ni, W, and O, revealing an atomic percentage of 15.42%, 16.02%, and 68.06%, respectively. The effective synthesis of stoichiometric NiWO_4_ on the SS substrate is confirmed by this composition. The EDS analysis of rGO-NiWO_4_ (Figure 2i) shows atomic percentage of Ni (12.30%), W (12.08%), O (57.59%), and C (18.03%), indicating formation of composite material.

**FIGURE 3:**
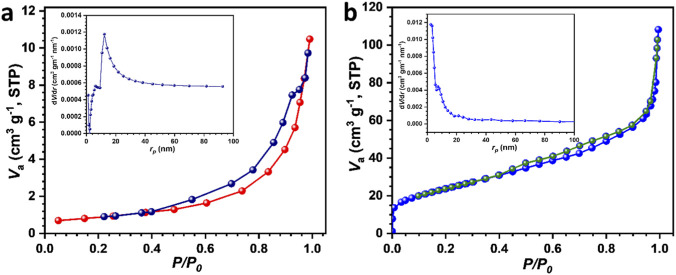
N_2_-sorption isotherms of **(a)** NiWO_4_ and **(b)** rGO-NiWO_4_ composite material (inset shows pore size distribution of respective materials).

The Brunauer-Emmette-Teller (BET) technique was utilized to analyze the effect of composition on surface area after rGO introduction. The nitrogen absorption isotherms for NiWO_4_ and rGO-NiWO_4_ composite materials are shown in Figure 3. The specific surface area of rGO-NiWO_4_ composite (63 m^2^ g^−1^) is higher than the NiWO_4_ (23 m^2^ g^−1^). This increase in surface area is associated with rGO composition. The pore size distribution of NiWO_4_ and rGO-NiWO_4_ composite materials, as inset of respective plot in Figure 3, exhibits a shift toward smaller pore sizes. This mesoporous nature is highly beneficial for catalytic applications, as it enhances the density of active sites, thereby improving overall catalytic performance (Abakumov et al., 2024; Xiao et al., 2021).

**FIGURE 4:**
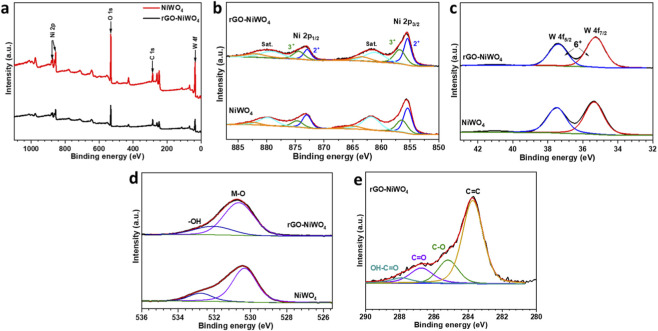
**(a)** Wide scan XPS spectrum, high-resolution XPS spectra of the **(b)** Ni 2p core level, **(c)** W 4f core level, **(d)** O 1s core level for NiWO_4_ and rGO-NiWO_4_ composite, and **(e)** C 1s core level from rGO-NiWO_4_ composite.

The electrocatalytic activity of the material is significantly influenced by the oxidation states of its constituent elements. To further analyze the surface composition of NiWO_4_ and rGO-NiWO_4_ composite material, XPS study was conducted to illustrate the surface valence states of elements. Both materials do not show large variation in the peak positions of the constituent elements, indicating formation of composite with little interaction between materials. The survey spectrum of NiWO_4_ and rGO-NiWO_4_ composite shown in Figure 4a exhibits the peaks corresponding to Ni 2p, W 4f, O 1s, and C 1s, confirming the presences and formation of composite material. The wide spectra with deconvolution of Ni 2p spectrum (Figure 4b) reveals two spin-orbit coupling pairs, Ni 2p_3/2_ and Ni 2p_1/2_, at binding energies of 855.6 and 873.34 eV, respectively. In addition to these peaks two satellite peaks are also observed in Ni 2p spectrum at energies of 879.4 eV and 861.6 eV, confirming a +2 oxidation state for Ni. The wide spectra of W4f (Figure 4c) display peaks at 37.45 eV for W 4f_5/2_ and 35.30 eV for W 4f_3/2_, with an energy separation of 2.15 eV, indicating the presence of W^6+^. The high resolution XPS spectrum of O 1s (Figure 4d) reveals a prominent peak at 531.07 eV, which corresponds to lattice oxygen, along with a smaller peak at 533.2 eV that can be attributed to oxygen from adsorbed water molecules. The C 1s spectrum form rGO-NiWO_4_ composite (Figure 4e) shows three well-defined peaks at 284.8 eV, 286.3 eV, and 288.2 eV. The peak at 284.8 eV is characteristic of sp^2^-hybridized carbon, confirming the presence of graphene-like structures. The signal at 286.3 eV arises from C–OH and C–O groups, while the peak at 288.2 eV is linked to O–C=O functional groups, highlighting the chemical diversity within the material.

**FIGURE 5:**
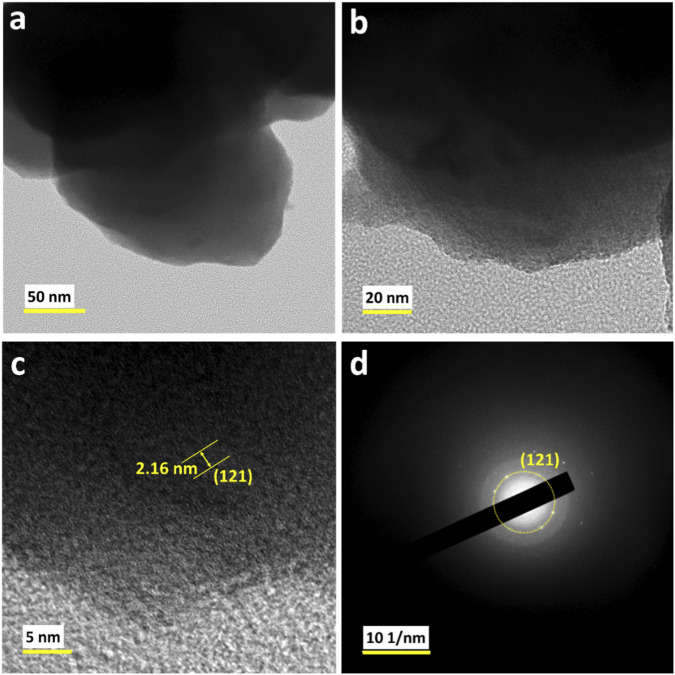
**(a)** TEM image, **(b,c)** HR-TEM, and **(d)** corresponding SAED pattern of rGO-NiWO_4_ composite.

The TEM analysis of rGO-NiWO_4_ composite shown in Figure 5a, reveals spherical nanoparticles as observed in the FE-SEM images. The HR-TEM images (Figures 5b,c) further confirm the observation from the XRD that the material has low crystalline nature. The line pattern observed for material is assigned to the (121) plane of NiWO_4_ and correspond to the ring pattern observed in the selected area electron diffraction (SAED) pattern (Figure 5d), consistence with the XRD data.

### 3.3 Electrochemical properties

**FIGURE 6:**
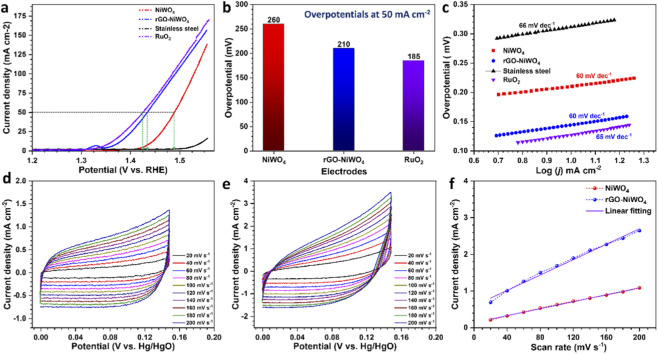
**(a)** The LSV curves for NiWO_4_, RuO_2_, SS, and rGO-NiWO_4_ composite. **(b)** overpotential values for NiWO_4_, RuO_2_, and rGO-NiWO_4_ composite, **(c)** Tafel plots for NiWO_4_, RuO_2_, SS, and rGO-NiWO_4_ composites. The CV curves in non-Faradaic potential for **(d)** NiWO_4_ and **(e)** rGO-NiWO_4_ composites at different scan rates, and **(f)** Plot of current density against scan rate for NiWO_4_ and rGO-NiWO_4_ composites.

The OER performance of NiWO_4_ and rGO-NiWO_4_ composite electrodes was evaluated using LSV measured at a scan rate of 1 mV s^−1^ in a 1 M KOH electrolyte (Figure 6a). The NiWO_4_ electrode exhibited a high overpotential of 260 ± 13 mV at a current density of 50 mA cm^−2^ compared to that of rGO-NiWO_4_ composite electrode demonstrated a significantly lower overpotential of 210 ± 10 mV, suggesting improved electrocatalytic activity. The LSV curves obtained for the SS substrate and RuO_2_-coated SS substrate indicate that rGO-NiWO_4_ composite electrode exhibits a lower overpotential compared to the uncoated SS substrate, though slightly higher than that of RuO_2_-coated SS (185 ± 2 mV). The improved electrocatalytic performance of rGO-NiWO_4_ composite electrode may be attributed to its increased surface area, the efficient charge transfer facilitated by the conductive network of rGO. The overpotential of the materials studied is plotted in Figure 6b. The reaction kinetics of the oxygen evolution reaction (OER) were evaluated through Tafel slope analysis (Figure 6c). The rGO–NiWO_4_ composite electrode exhibited a Tafel slope of 60 ± 3 mV dec^−1^, which is comparable to that of pure NiWO_4_ and lower than the values obtained for the stainless steel (SS) substrate (66 ± 3 mV dec^−1^) and RuO_2_-coated SS (65 ± 3 mV dec^−1^). This reduction in Tafel slope reflects faster reaction kinetics and improved electron transfer, highlighting the superior catalytic performance of the rGO–NiWO_4_ composite. The CV curves recorded at a scan rate of 50 mV s^−1^ (Supplementary Figure S3) show no significant change in the positions of the oxidation and reduction peaks. However, the current density at higher potentials varies among the samples, possibly due to differences in the composition and electrical conductivity of the materials.

**FIGURE 7:**
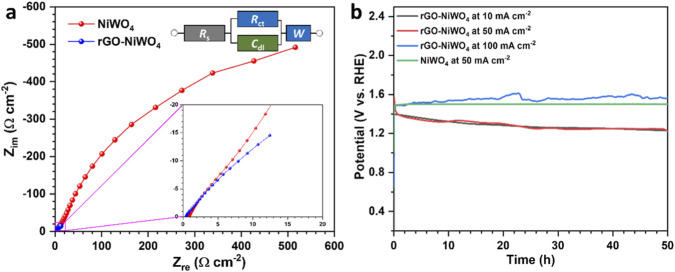
**(a)** Nyquist plots of NiWO_4_, rGO-NiWO_4_ composite electrode, and **(b)** stability plots of the NiWO_4_ and rGO-NiWO_4_ composite electrode assessed at different current densities.

To gain insights into cause of improvement in the electrocatalytic performance for OER, the ECSA measurement was estimated. For this, the CV measurements were conducted at varying scan rates as shown in Figures 6d,e, and *C*_dl_ was estimated from the slope of the current density vs. scan rate plot (Figure 6f). rGO-NiWO_4_ composite electrode exhibited a *C*_dl_ value of 10.5 ± 0.5 mF cm^−2^, approximately two times higher than that of NiWO_4_ (4.7 ± 0.25 mF cm^−2^), indicating a higher density of electrochemically active sites. The ECSA for NiWO_4_ and rGO-NiWO_4_ composite electrode are 117.5 ± 6 cm^2^ and 262.5 ± 13 cm^2^, respectively. The increased ECSA value for rGO-NiWO_4_ composite electrode can be attributed to the layered architecture and higher surface area formed by rGO composition, which facilitates enhanced electrolyte accessibility. Furthermore, to assess the impact of rGO incorporation on charge transfer, the EIS measurements were conducted, and the Nyquist plot is presented in Figure 7a. The results demonstrate a notably lower *R*_ct_ for rGO-NiWO_4_ composite electrode (6.8 Ω) in comparison to NiWO_4_ (389 Ω), indicating enhanced interfacial charge transfer in the composite material. Additional parameters, including solution resistance (*R*_s_), Warburg impedance (*W*), and *C*_dl_, for both electrodes are summarized in Table 1. Corresponding Equivalent circuit is provided in the inset of Figure 7a. Considering the superior performance exhibited by the rGO-NiWO_4_ composite electrode, its long-term electrochemical stability was assessed using chronopotentiometry under a fixed current density of 50 mA cm^−2^ for 50 h (Figure 7b). The rGO-NiWO_4_ composite electrode displayed remarkable stability, maintaining a consistent potential over the entire test duration. This study suggests that the electrocatalyst effectively preserves its active sites under extended electrochemical conditions.

**TABLE 1:** Impedance parameters of NiWO_4_ and rGO-NiWO_4_ of electrodes.

Electrode	*R*_s_ (Ω)	*R*_ct_ (Ω)	*C*_dl_ (F)	*W* (Ω)	*χ* ^*2*^
NiWO_4_	0.92	389	0.0824	0.00012	0.9899
rGO-NiWO_4_	0.80	6.80	0.0951	0.0589	0.9912

## 4 Conclusion

The development of rGO/NiWO_4_ electrocatalysts via the SILAR method demonstrates significant advancements in OER performance for water splitting applications. While NiWO_4_ exhibited limited OER activity, the integration of rGO notably enhanced the catalytic performance. The rGO/NiWO_4_ composite electrode showed a lower overpotential and Tafel slope (210 ± 10 mV, 60 ± 3 mV dec^−1^) and reduced charge transfer resistance. Moreover, the outstanding stability of the composite electrode, maintaining stable potential over 50 h of continuous operation, underscore its potential as a promising electrocatalyst. These findings highlight rGO/NiWO_4_ as a highly effective material for sustainable OER and renewable energy applications, with considerable implications for advancing water splitting technologies.

## Data Availability

The original contributions presented in the study are included in the article/Supplementary Material, further inquiries can be directed to the corresponding author.
