# Supplementary material for: Facile synthesis of rGO/NiWO4 hybrid electrocatalyst for enhanced oxygen evolution reaction in alkaline medium
Source: Front Chem. 2026 Apr 28;14:1799700. doi: 10.3389/fchem.2026.1799700 (PMC13161117; doi:10.3389/fchem.2026.1799700)
Supplement: Supplementary file 1 [file DataSheet1.docx]

**Facile Synthesis of rGO/NiWO_4_ Hybrid Electrocatalyst for Enhanced Oxygen Evolution Reaction in Alkaline Medium**

*D. J. Patil^1,2✝^, D. B. Malavekar**^3✝^, V.C. Lokhande^4^, J. H. Kim^3^, and C.D. Lokhande^1^**

**^1^**Centre for Interdisciplinary Research, D. Y. Patil Education Society (Deemed to Be University), Kolhapur 416 006, India

^2^Department of Applied Science and Humanities, TKIET, Warana University Warananagar, Kolhapur 416114, India

^3^Department of Materials Science and Engineering, Chonnam National University, Gwangju, 61186, South Korea.

^4^Energy storage research group, School of Chemistry and Physics, Queensland University of Technology Brisbane, Australia - 4001

**S1. Characterizations**

The structural and compositional properties of the deposited materials were examined using a range of advanced characterization techniques. The crystal structure was analysed with an X-ray diffractometer (Rigaku MiniFlex-600, Japan) operating with CuKα radiation (λ = 1.54 Å). To identify functional group interactions, Fourier transform infrared (FT-IR) spectroscopy was carried out using a Bruker Tensor 27 instrument (United States). Raman spectra were recorded with a JASCO NRS-5100 spectrometer (Japan) at an excitation wavelength of 532 nm. Surface morphology and topographical features were observed using a field emission scanning electron microscope (FE-SEM) (Model: JEOL JEM 2100, Japan). The oxidation states and elemental composition of the electrode material were determined through X-ray photoelectron spectroscopy (XPS) using a Kα^+^ system (Thermo Fisher Scientific, United States) equipped with a monochromatic Mg Kα source (1253.6 eV). High-resolution transmission electron microscopy (HR-TEM) measurements were performed with a JEOL ARM-200F field emission TEM (Japan) to gain deeper insights into the nanostructure. Finally, the specific surface area and pore size distribution were evaluated using Brunauer–Emmett–Teller (BET) and Barrett–Joyner–Halenda (BJH) models, respectively, with Quantachrome Instruments software (v11.02.2.4, Austria). The BET measurements were carried out using powder obtained by scratching the deposited film from the SS substrate after film formation. Approximately 0.15 g of the collected powder was used for the BET analysis. Prior to the measurements, the sample was degassed at 120 ℃ for 9 h to remove physically adsorbed moisture and gases.

Electrochemical analyses of electrocatalysts were conducted using a Zive MP1 electrochemical potentiostat (WonATech Co., Ltd., South Korea) in a standard three-electrode setup. The reference electrode was a mercury/mercury oxide (Hg/HgO) electrode, while a platinum sheet of 5 cm^2^ acted as the counter electrode. The working electrode comprised of NiWO_4_ and rGO-NiWO_4_ materials. The linear sweep voltammetry (LSV) was conducted at various potential scan rate of 1 mV s^−1^ within the range of +0.1 V to +0.85 V vs. Hg/HgO in a 1 M KOH electrolyte solution. The measured potential values were converted from Hg/HgO scale to the reversible hydrogen electrode (RHE) scale using the Nernst equation:

E_RHE_ = E^0^ + 0:059 x pH + E_Hg/HgO_ (1)

where 𝐸_Hg/HgO_ is the measured potential against the Hg/HgO electrode, 𝐸_RHE_ is the converted potential against RHE, and 𝐸^0^ is the standard redox potential of Hg/HgO at 298 K (0.140 V ± 0.001 V). 85% *iR* correction was applied for LSV measurements for overpotential calculation. The electrocatalytic efficiency in terms of overpotential (𝜂) was calculated from the LSV curve at a current density (j) using the following formula:

η_@j_ (V) = E_RHE_ (V) – 1.23 (V) (2)

The Tafel slope was determined using equation (3), which was obtained from the linear fit of the Tafel plot:

η = b log j + a (3)

In this case, 𝑎 is the fitting parameter and 𝑏 (mV dec^−1^) is the Tafel slope. The electrocatalytic stability of the OER electrodes was assessed using chronopotentiometry measurement at 50 mA cm^−2^ over 50 hours. Cyclic voltammograms (CV) were used to quantify the electrochemical double-layer capacitance (*C*_dl_) in the potential range of 0.15 to 0.25 V vs. Hg/HgO at different scan rates (between 20 and 100 mV s^−1^). These Cdl values were then used to calculate the electrochemically active surface area (ECSA) using the following formula:

ECSA = *C*_dl_/0:04 (4)

The figure of 0.04 mF cm^-2^ represents the average specific capacitance of a standard flat surface with an area of 1 cm^2^ in an alkaline atmosphere. Electrochemical impedance spectroscopy (EIS) was used to determine resistive properties of the electrocatalysts. These measurements were performed at an alternating current (AC) signal with a 10 mV amplitude throughout a frequency range of 100 mHz to 100 kHz.

**Figure S1.** Raman spectra of rGO.


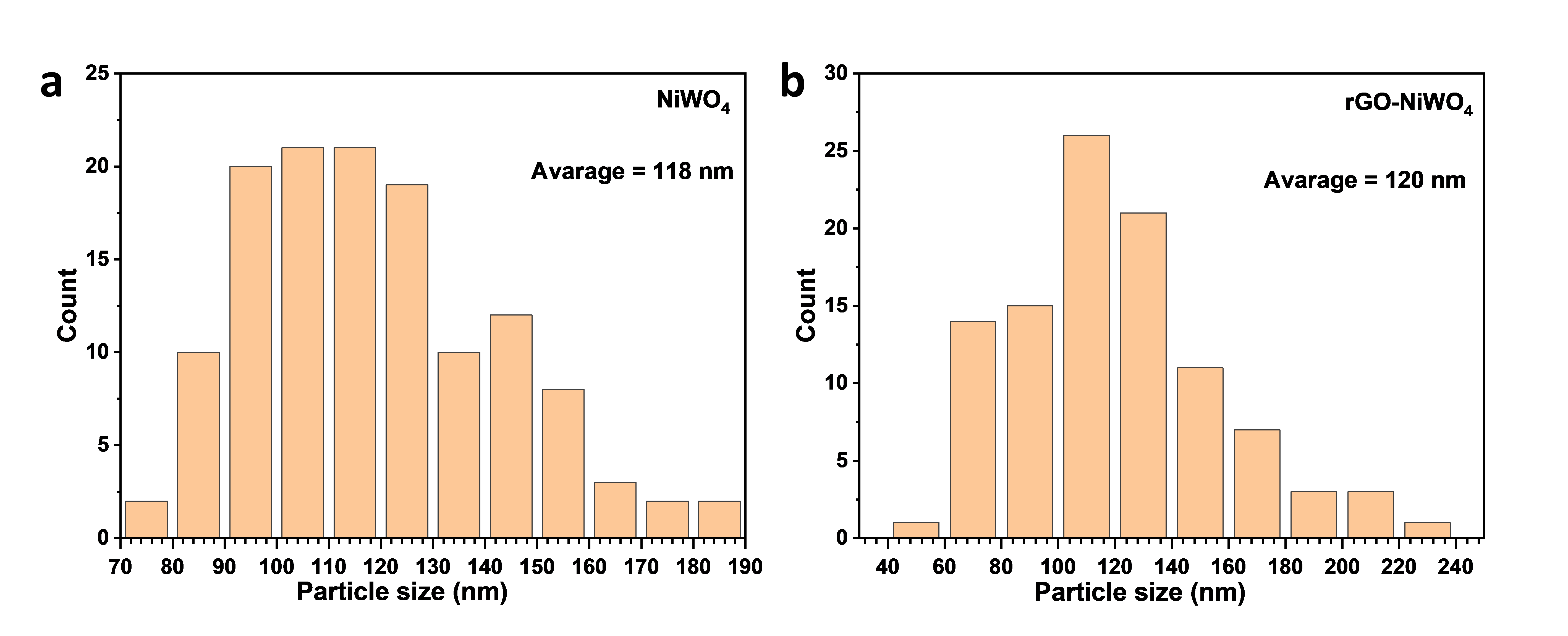


**Figure S2.** Particle size distribution of NiWO_4_ and rGO-NiWO_4_ obtained from SEM images.

**Figure S3.** CV curves of NiWO_4_ and rGO-NiWO_4_ catalyst measured at 50 mV s^−1^ scan rate.
